# The compensatory dynamic of inter-hemispheric interactions in visuospatial attention revealed using rTMS and fMRI

**DOI:** 10.3389/fnhum.2014.00226

**Published:** 2014-04-17

**Authors:** Ela B. Plow, Zaira Cattaneo, Thomas A. Carlson, George A. Alvarez, Alvaro Pascual-Leone, Lorella Battelli

**Affiliations:** ^1^Department of Biomedical Engineering and Department of Physical Medicine and Rehabilitation, Cleveland ClinicCleveland, OH, USA; ^2^Department of Neurology, Beth Israel Deaconess Medical Center, Berenson-Allen Center for Noninvasive Brain Stimulation, Harvard Medical SchoolBoston, MA, USA; ^3^Department of Psychology, University of Milano-BicoccaMilano, Italy; ^4^Brain Connectivity Center, National Neurological Institute C. MondinoPavia, Italy; ^5^Department of Cognitive Science, Macquarie UniversitySydney, NSW, Australia; ^6^Department of Psychology, Harvard UniversityCambridge, MA, USA; ^7^Instituto Guttmann de Neurorrehabilitación, Universidad Autónoma de BarcelonaBadalona, España; ^8^Center for Neuroscience and Cognitive Systems@UniTn, Fondazione Istituto Italiano di TecnologiaRovereto, Italy

**Keywords:** visual extinction, inter-hemispheric interaction, visuospatial attention, TMS, fMRI

## Abstract

A balance of mutual tonic inhibition between bi-hemispheric posterior parietal cortices is believed to play an important role in bilateral visual attention. However, experimental support for this notion has been mainly drawn from clinical models of unilateral damage. We have previously shown that low-frequency repetitive TMS (rTMS) over the intraparietal sulcus (IPS) generates a contralateral attentional deficit in bilateral visual tracking. Here, we used functional magnetic resonance imaging (fMRI) to study whether rTMS temporarily disrupts the inter-hemispheric balance between bilateral IPS in visual attention. Following application of 1 Hz rTMS over the left IPS, subjects performed a bilateral visual tracking task while their brain activity was recorded using fMRI. Behaviorally, tracking accuracy was reduced immediately following rTMS. Areas ventro-lateral to left IPS, including inferior parietal lobule (IPL), lateral IPS (LIPS), and middle occipital gyrus (MoG), showed decreased activity following rTMS, while dorsomedial areas, such as Superior Parietal Lobule (SPL), Superior occipital gyrus (SoG), and lingual gyrus, as well as middle temporal areas (MT+), showed higher activity. The brain activity of the homologues of these regions in the un-stimulated, right hemisphere was reversed. Interestingly, the evolution of network-wide activation related to attentional behavior following rTMS showed that activation of most occipital synergists adaptively compensated for contralateral and ipsilateral decrement after rTMS, while activation of parietal synergists, and SoG remained competing. This pattern of ipsilateral and contralateral activations empirically supports the hypothesized loss of inter-hemispheric balance that underlies clinical manifestation of visual attentional extinction.

## Introduction

Visual attention depends upon the balance of tonic inhibition exerted between bilateral posterior parietal cortices (Kinsbourne, 1977; Muri et al., 2002; Battelli et al., 2009). Unilateral lesions can disrupt this balance, resulting in visual extinction, the inability to perceive contra-lesional targets when competing targets are presented bilaterally (Vallar et al., 1994). One hypothesis is that the damaged posterior parietal cortex is unable to “compete” against the uninhibited activity of the intact homologue, which in turn hyper-orients attention to the ipsi-lesional visual field leading to extinction of targets in the contra-lesional space (Kinsbourne, 1977). Still, *direct* evidence of inter-hemispheric competition in posterior parietal cortices is limited. Inferences have been drawn from clinical neuropsychological observations (Battelli et al., 2001; Corbetta et al., 2005). However, this approach is severely limited because lesions have widespread, unpredictable effects that make it challenging to disentangle their direct sequel from ensuing disruptions in inter-hemispheric balance (Pascual-Leone et al., 2005).

Neurophysiological techniques can help determine the cause-effect relation between activity of posterior parietal cortices and behavior more reliably (Pascual-Leone et al., 1999; Walsh and Cowey, 2000) and, to this aim, repetitive transcranial magnetic stimulation (rTMS) can transiently disrupt activity in targeted posterior parietal cortex and induce reversible behavioral impairments (Hilgetag et al., 2001; Muri et al., 2002; Thut et al., 2005; Dambeck et al., 2006; Fierro et al., 2006; Battelli et al., 2009). For instance, we have previously used rTMS to study inter-hemispheric balance between bilateral posterior parietal cortices in sustained visual attention (Battelli et al., 2009). Transient disruption of unilateral posterior intra-parietal sulcus (IPS) with low-frequency rTMS worsened contra-lateral visual attention. Since this effect only manifested during a task that required bilateral attention, simulating “visual extinction” (Vallar et al., 1994), we hypothesized that rTMS disrupted the balance of inter-hemispheric inhibition exerted between bilateral IPS during full-field attention (Battelli et al., 2009).

However, since effects of rTMS are not limited to the directly targeted region, but instead they may also affect distant cortical and subcortical structures (Fox et al., 2012), ascribing behavioral impairments to inter-hemispheric competition between homologous pairs of IPS based on use of rTMS alone was only speculative. It still remains unclear whether the impairment was caused by disruption of activity of the directly targeted cortical locus (IPS), or its competition with its homologue, or if in fact TMS temporarily altered the network-wide balance between all areas involved in sustained attention (Ruff et al., 2008; Blankenburg et al., 2010).

Recent evidence combining TMS with functional neuroimaging suggests that TMS may have causal influence that extends beyond the targeted locus, involving remote synergists as well as homologous and heterologous regions in the non-targeted hemisphere (Blankenburg et al., 2010). We thus posited that examining the effects of TMS with functional neuroimaging would clarify our speculations regarding network-wide effects of TMS (Ruff et al., 2008; Fox et al., 2012).

To gather *direct* evidence that extinction induced with low-frequency rTMS targeting IPS evolves from modulated inter-hemispheric balance, we created a new empirical design as a follow up to our previous protocol. Briefly, low-frequency 1 Hz rTMS or sham was delivered to the left IPS, promptly followed by 3 experimental runs of functional magnetic resonance imaging (fMRI) while subjects performed sustained bilateral attention involving visual tracking. We chose bilateral visual attention since recent fMRI studies have demonstrated that when stimuli presented in both right and left hemifields are task relevant, requiring high attentional competition, then left and right IPS are equally activated (Geng et al., 2006). Disruption of IPS prior to bilateral visual attention, we believed, would amplify the inter-hemispheric imbalance we note with fMRI activation. We chose to deliver low-frequency rTMS to left IPS because we were extending our previous results where experimentally induced extinction was greater with rTMS to the left than to the right IPS (Battelli et al., 2009). Still, we used a slightly different visual stimulus than in our previous study (Carlson et al., 2007; Battelli et al., 2009) because the present stimulus was more likely to elicit fMRI activation in early visual areas. Their study in network-wide effect was important as they are strongly influenced by attention (Somers et al., 1999).

Overall, we hypothesized that our protocol of rTMS-induced contralateral visual extinction, when studied with fMRI, would potentially demonstrate: (a) reduced fMRI activation of targeted IPS with exaggerated activation of its homologue, indicating disrupted inter-hemispheric balance underlying extinction, (b) besides IPS, a network-wide shift in inter-hemispheric activation of areas involved in sustained visual attention, which would demonstrate that extinction involves synergistic areas extending beyond targeted IPS and (c) an association between extinction and its alleviation with evolution of activation of synergists would reveal the type of functional role they exert in supporting IPS sustain bilateral attention. Study of an experimental protocol examining extinction rather than neglect is significant because while visual neglect is known to result from right parietal lesion (Battelli et al., 2001; Mort et al., 2003), lesions underlying visual extinction are less clear (Stone et al., 1993).

## Materials and methods

### Subjects

Ten healthy subjects (mean age ± *SD* 27.72 ± 5.99 years, 7 males) participated in the experiment; one subject was excluded from analysis due to excessive head motion in the MRI scanner. All subjects had normal or corrected-to-normal vision. All participants met all TMS (Rossi et al., 2009) and MRI screening criteria and provided written informed consent in accordance with the Institutional Review Board of the Beth Israel Deaconess Medical Center, Boston, MA.

### Behavioral task

**Figure 1:**
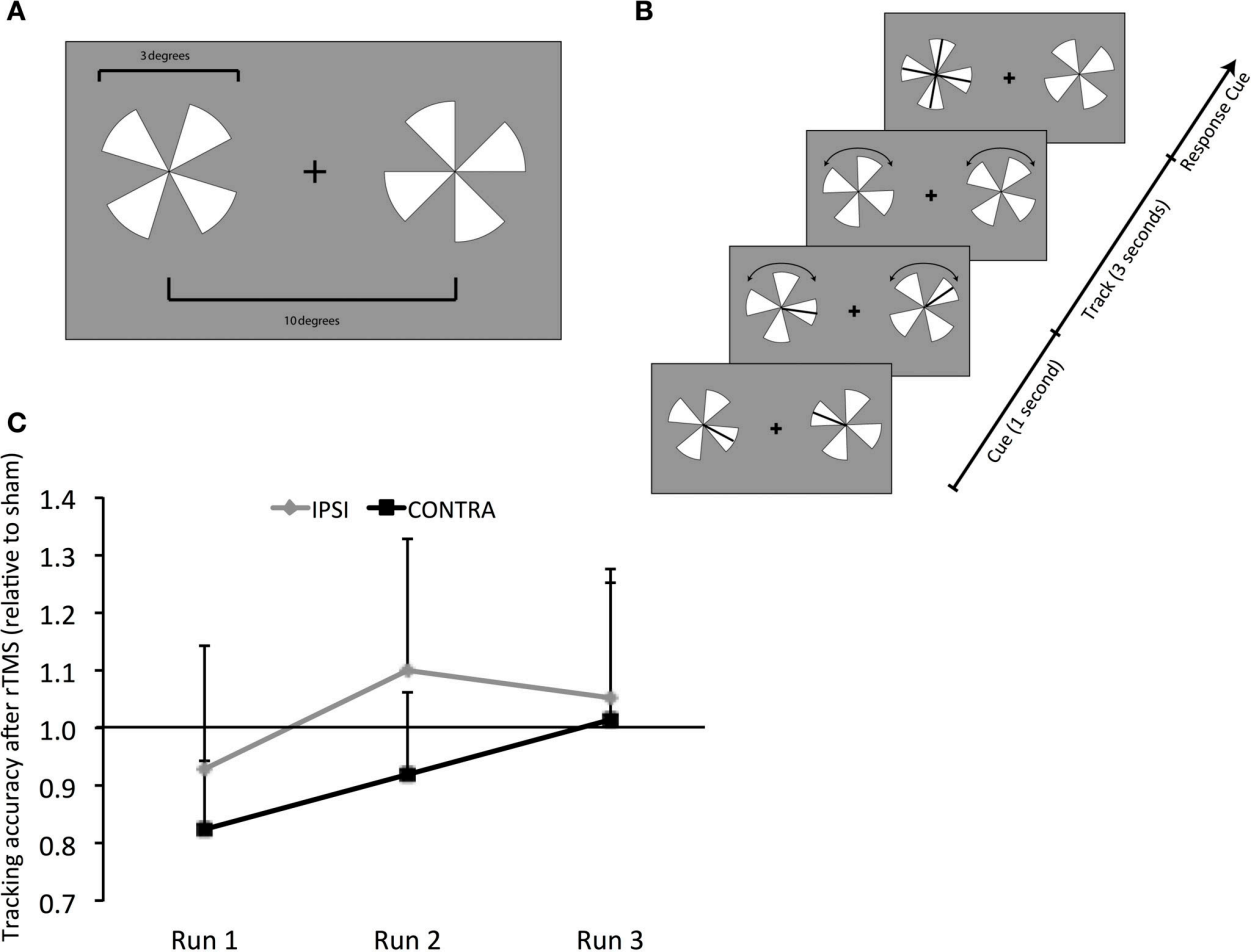
**Behavioral Task - Design and Results. (A)** Visual tracking task. Stimuli were high-contrast pairs of pinwheels displayed on either side of a central fixation cross. **(B)** At the start, the targets (a randomly selected spoke on each pinwheel) were cued briefly. Following the cues disappearance, the pinwheels rotated at a fixed rate, determined by individual subjects' threshold for 85% correct performance, while subjects tracked the targets. After 3 s, both pinwheels stopped and were aligned so that all probes on the target pinwheel appeared as a cross. Subjects responded using a four-alternative forced-choice paradigm (“up,” “down,” “left,” or “right” keys) to report which of the probes represented the originally-cued target. **(C)** Results of tracking accuracy: contra, contralateral (right hemifield); ipsi, ipsilateral (left hemifield); y-axis, tracking accuracy following rTMS as a proportion of that following sham; x-axis, experimental runs (1 through 3). Values below 1 represent a decrement and above 1 show improvement following rTMS vs. sham.

Each subject participated in a total of six experimental runs simultaneous with fMRI- three following rTMS and three following sham conducted on 2 separate days in a counter-balanced order. During each fMRI run, subjects performed bilateral visual tracking (Figures 1A,B) as in our previous work (Carlson et al., 2007; Battelli et al., 2009). Bilateral visual tracking typically involves sustained attention, a well-studied behavioral paradigm with clear neural correlates (Culham et al., 1998; Battelli et al., 2001; Drew and Vogel, 2008). In this task, high-contrast pairs of pinwheels were displayed on either side of central fixation. On each pinwheel, targets were represented as a randomly selected spoke cued briefly. Following disappearance of cues, both pinwheels rotated at a fixed rate, pre-determined by individual's threshold for performing at 85% accuracy. Subjects tracked the target spokes bilaterally for 3 s, after which the pinwheels stopped. When they stopped, the pinwheels were displayed upright. All spokes re-appeared as probes (in the form of a cross) on the target pinwheel. Subjects were asked to respond using a four-alternative forced-choice key-press (“up,” “down,” “left,” or “right” keys) which probe represented the originally cued target spoke. Stimuli were generated in MATLAB using functions of the Psychophysics Toolbox (Brainard, 1997; Pelli, 1997) and displayed on a PC laptop with a 17” monitor screen projected with a rear-view mirror attached to the head coil in the scanner. A total of 35 trials were presented for each experimental run.

### rTMS

TMS was applied using a MagStim device (MagStim, Whitland, Wales, UK) with a 70-mm figure-of-eight coil. It was guided by neuronavigation to the individually defined left IPS and it was calculated as the average MRI-defined stereotaxic coordinates from our prior study (Battelli et al., 2009) [Talairach (mean ± *SD*): *X* (−23.37 ± 5.24), *Y* (−67.60 ± 4.25) and *Z* (52.88 ± 2.47) mm]. These pre-defined coordinates were translated into individual's native brain space using frameless stereotaxic image guidance (Brainsight™, Rogue Research Inc., Montreal, QC, Canada). The TMS coil was held with the handle pointing posteriorly at an angle of 45° to the inter-hemispheric fissure, at an orientation that aligned it perpendicular to the left IPS. Low-frequency 1 Hz rTMS was applied for 15-min at 75% of the maximum stimulator output. For the sham condition we placed the edge of the coil at an angle perpendicular to the head, while stimulation was delivered at the same intensity as in the rTMS session. Experimental runs involving bilateral visual tracking concurrent with fMRI were initiated within four minutes from completion of rTMS/sham.

### fMRI

MRI and Blood Oxygen Level-Dependent (BOLD) fMRI data was acquired in a whole-body 3T Phillips scanner equipped with 22 mT/m field gradients with a slew rate of 120 T/m/s. FMRI scan parameters were: TR = 2 s, TE = 55 ms, flip angle = 90°, imaging matrix = 96 × 96, FOV = 23 cm and 20 slices. Slices were 4 mm thick with an in-plane resolution of 2.4 × 2.4 mm and a gap of 0.5 mm. Gradient-echo planar imaging (EPI) sequence was used with a standard head coil. Structural MRI data was collected in an MPRAGE high resolution, *T*_1_-weighted format in sagittal orientation. The total number of slices sampled was 170. Parameters of MPRAGE data were: FOV- 240 × 256 × 204 mm; TR = 7 ms; spatial resolution- 1 × 1 × 1.2 mm with no gap.

### Data and statistical analysis

#### Behavior

Behavioral accuracy was computed as percent correct response. For each run, percentage accuracy following rTMS was normalized to that following corresponding sham run. Values below 1 indicate impairment following rTMS while those above 1 indicate improvement. This normalized accuracy value was compared between contralateral (right) and ipsilateral (left) hemifields, and between one run and another using pairwise, within-group comparisons (using Student's *t*-test).

#### fMRI

Analysis was conducted using Brain Voyager QX 1.10 (Brain Innovation, Maastricht, Netherlands). Functional data was preprocessed for 3-D motion correction (Cox and Hyde, 1997), removal of temporal linear trends, and correction for slice time acquisition and then spatially smoothed (Gaussian kernel, 3.0 mm FWHM). Individual subjects' data was normalized to Talairach space (Goebel et al., 2006). A single-factor design matrix was generated including the predictor of interest, visual tracking. The predictor was derived by convolving a box-car waveform with a double-gamma hemodynamic response function (Friston et al., 1999). General Linear Model (GLM) was applied to time series data for each subject and a statistical map of “bilateral visual attentional tracking” and its “variability” was computed.

**Figure 2:**
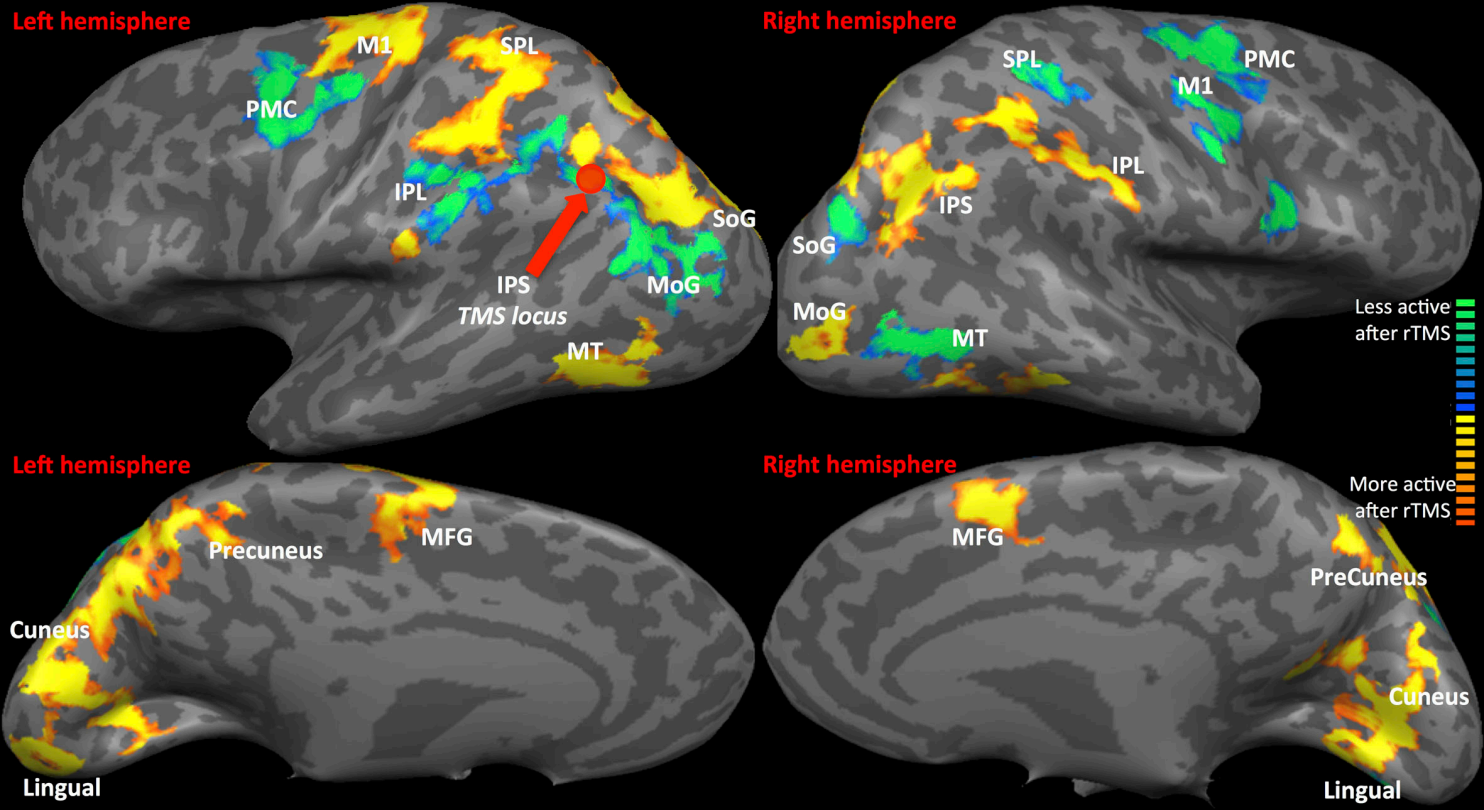
**fMRI-Regions of interest analysis (ROIs) identified from Multi-Subject Fixed Effects GLM analysis.** FMRI Activation Maps displaying results of Multi-subject fixed effects GLM with comparisons between pooled runs (1, 2, and 3) of rTMS vs. sham. Locus of rTMS targeting is shown as filled purple circle over the left IPS. Red-to-yellow: activation following rTMS > sham; blue-to-green: activation following rTMS < sham. Abbreviations are expanded in Table 1.

**Figure 3:**
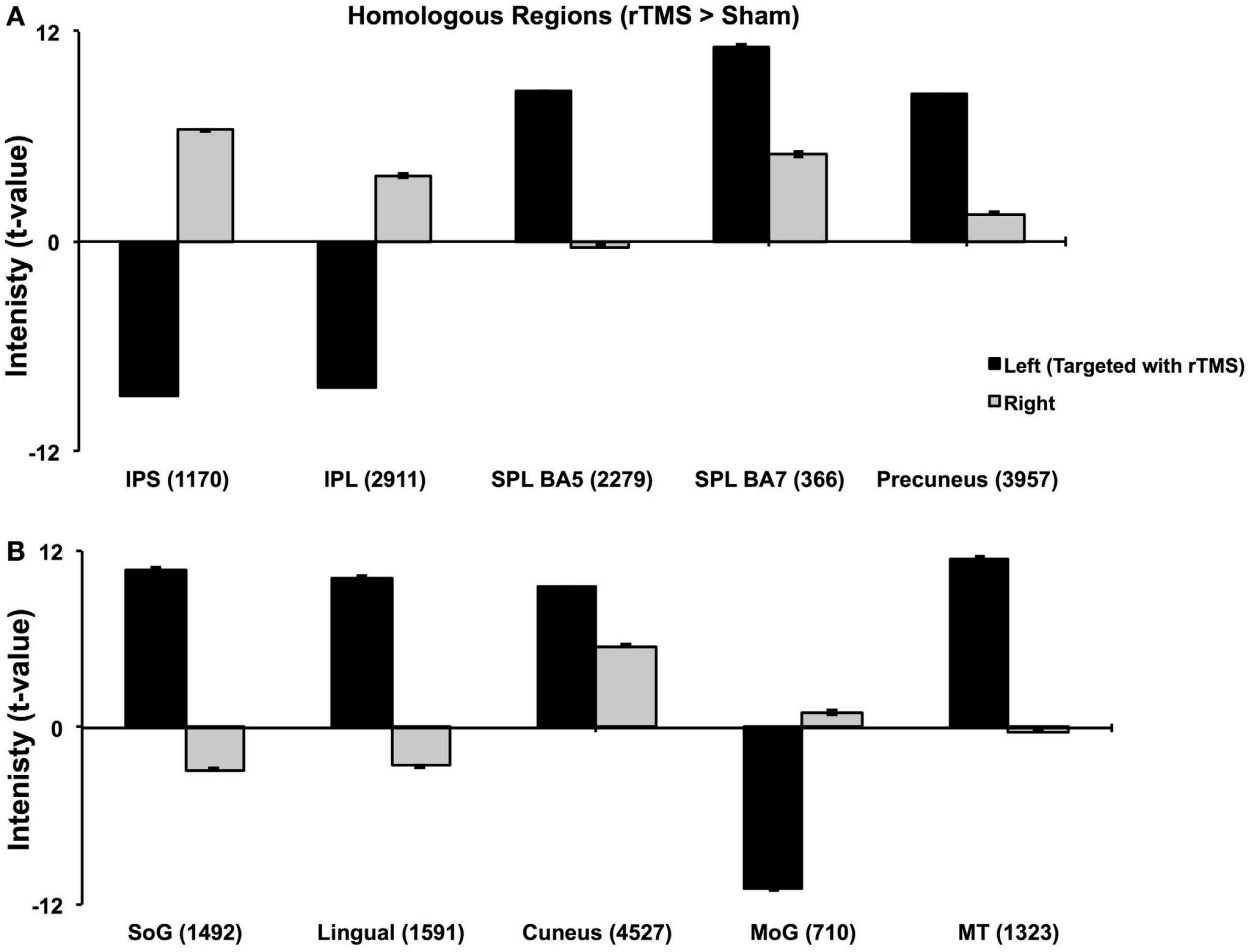
**Analysis of Homologous ROIs.** Quantitative comparison of *t*-values of intensities of homologous ROIs. For homologous ROIs of comparable size (number of voxels noted in parentheses), *t*-values of intensity on left (targeted hemisphere) are compared with those of their homologues on right. **(A)** Comparison of homologous voxels pairs in parietal lobes. **(B)** Comparison of homologous voxels pairs in occipital and temporal regions. While IPS, IPL and MoG show lower intensities in targeted hemisphere, SPL, SoG, Lingual and MT+ show higher intensities in targeted than right hemisphere. These results confirm findings in Figure 2.

**Figure 4:**
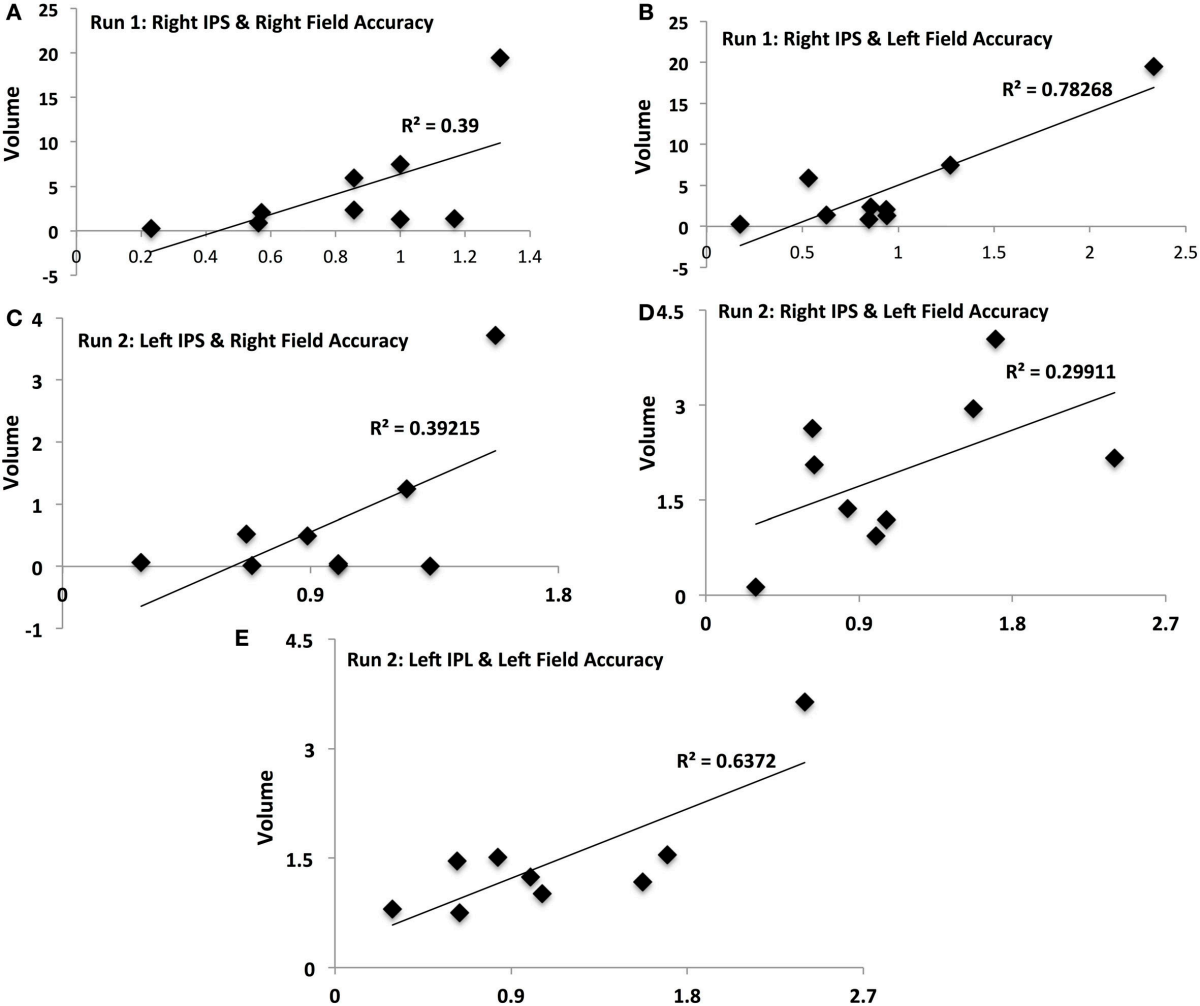
**Activation-accuracy relationships for IPS and Parietal regions.** Figure shows relationship between accuracy in contralateral and ipsilateral fields in runs 1 and 2 and volumes of activation of parietal ROIs. Note that accuracy in each hemifield is represented as accuracy following rTMS normalized to that following sham. Similarly, volume of activation of an ROI or its intensity is computed as that following rTMS vs. that following sham. Pearson's *r* values are listed in Table 2. Relationships are denoted between **(A)** contralateral (Right Field) accuracy and activation of Right IPS in Run 1, **(B)** ipsilateral (Left Field) accuracy and activation of Right IPS in Run 1, **(C)** contralateral accuracy and activation of Left IPS in Run 2, **(D)** ipsilateral accuracy and activation of Right IPS in Run 2, and **(E)** ipsilateral accuracy and activation of Left IPL in Run 2.

**Figure 5:**
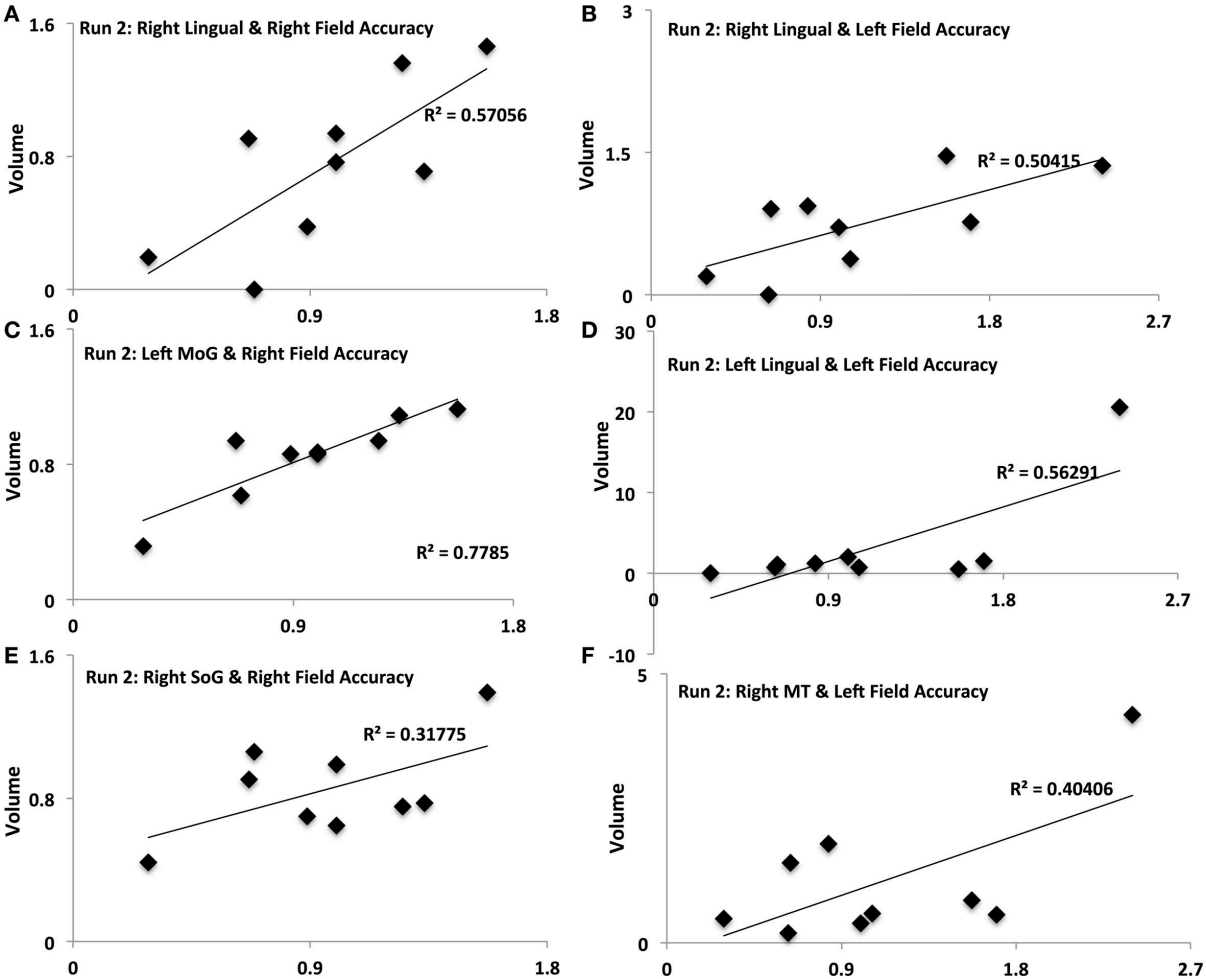
**Activation-accuracy relationships for other ROIs.** Figure shows relationship between accuracy in contralateral and ipsilateral fields in run 2 and volumes of activation of occipito-temporal ROIs. Note that accuracy in each hemifield is represented as accuracy following rTMS normalized to that following sham. Similarly, volume of activation of an ROI or its intensity is computed as that following rTMS vs. that following sham. Pearson's *r* values are listed in Table 2. Relationships are denoted between **(A)** contralateral (Right Field) accuracy and activation of Right Lingual in Run 2, **(B)** ipsilateral (Left Field) accuracy and activation of Right Lingual in Run 2, **(C)** contralateral accuracy and activation of Left MoG in Run 2, **(D)** ipsilateral accuracy and activation of Left Lingual in Run 2, **(E)** contralateral accuracy and activation of Right SoG in Run 2, and **(F)** ipsilateral accuracy and activation of Right MT in Run 2.

- *fMRI: Statistical Parametric map of rTMS vs. Sham (as displayed in Figure 2 and Table 1*) The contrast of visual tracking and its associated standard errors were included in a group (multi-subject) Fixed Effects GLM analysis (Soleymani et al., 2009) since it is sensitive for studies with a limited subject pool (Friston et al., 1999). The multi-subject GLM included all runs, and all 35 trials belonging to each run (1, 2, and 3), following rTMS and following sham. This GLM investigated whether greater signal change in bilateral tracking follows rTMS vs. sham. The GLM analysis yielded a statistical parametric map at a threshold of Bonferroni-corrected α = 0.01 with a spatial cluster threshold of 100 mm^3^. Figure 2 and Table 1 define its results demonstrating across the entire group of 10 subjects which areas show higher activation across pooled runs following rTMS than sham. Whereas red/yellow colors emphasize areas showing higher activity, blue-to-green colors indicate areas with lower activity following rTMS vs. sham (Figure 2). The overall map was classified into functional cortical regions of interest (ROIs) using Brodmann area (BA) nomenclature derived from Talairach localization (Talairach Daemon) (Lancaster et al., 2000).
- *fMRI- Analysis of homologous ROIs:* While multi-subject GLM gave an overview of differential activation of rTMS vs. sham across both hemispheres, we next compared intensities of voxels on the left vs. those on the right. For this, we chose to study ROIs from multi-subject analyses that were active in both hemispheres (Table 1). Voxels active in left ROI (targeted hemisphere) were mirrored on the right (sign for Talairach x-coordinate was reversed). We chose to mirror ROI from left upon right because left hemisphere had larger ROIs (Figure 2). From mirrored ROIs, for instance for mirrored pair of IPS, we identified “homologous” voxels, i.e., a common set of voxels that were significantly active across pooled runs in left as well as the right hemispheres. We compared *t*-values of intensity between voxels on the left with their homologues on the right using pairwise comparisons. For each pair, mean ± se of intensity is plotted in Figure 3.
- *fMRI: Activation-Accuracy correlation:* We next investigated activation of which ROIs ultimately relates to behavioral accuracy in each hemifield, and the chronology of such association. Exploring these serial effects is important because it could highlight the nature of the functional role exerted by a region in sustained attention. Unlike multi-subject (Figure 2) and homologous ROI analysis (Figures 3A,B) described above, where pooled runs were compared between rTMS and sham across all subjects, associations were analyzed separately for each run across individual subjects. ROIs defined by multi-subject analysis (Figure 2) were evaluated for each subject. We determined normalized activation of their ROI, i.e., volume of activation following each run of rTMS vs. corresponding run of sham. Their normalized accuracy- accuracy following each run of rTMS vs. corresponding run of sham (as in Figure 1)- was also noted. We computed the association (Pearson's correlation) between normalized accuracy within contralateral and ipsilateral hemifields and normalized activation of each ROI (Figure 4). We first examined correlations for IPS. Subsequently, as *post-hoc* exploratory analyses, we examined associations between accuracy and activation of other ROIs from multi-subject analysis (Figure 5).

**Table 1:** **fMRI-Regions of interest analysis (ROIs) identified from Multi-Subject Fixed Effects GLM analysis (Figure 2)**.

**Item**	**ROI**	**Volume**	***X***	***Y***	***z***	***t*-value sham**	***t*-value rTMS**
**rTMS > SHAM**							
1	L MFG BA6	2250	−12	−10	58	*t* = 28.03, *p* < 0.001	*t* = 44.54, *p* < 0.001
2	L M1 BA4	2107	−22	−21	62	*t* = −4.09, *p* = NS	*t* = 12.30, *p* < 0.001
3	L SPL BA5 cluster 1	2648	−27	−42	59	*t* = 16.84, *p* < 0.001	*t* = 33.23, *p* < 0.001
4	L SPL BA5 cluster 2	1851	−32	−38	43	*t* = 25.89, *p* < 0.001	*t* = 36.25, *p* < 0.001
5	L SoG BA19	1876	−24	−76	30	*t* = 41.74, *p* < 0.001	*t* = 57.64, *p* < 0.001
6	L Precuneus BA7	4923	−11	−69	49	*t* = 8.58, *p* < 0.001	*t* = 24.45, *p* < 0.001
7	L Cuneus BA18	5577	−7	−78	22	*t* = −27.79, *p* < 0.001	*t* = −7.47, *p* < 0.001
8	L Lingual BA17	2137	−6	−89	1	*t* = 13.82, *p* < 0.001	*t* = 30.18, *p* < 0.001
9	L MT	1819	−43	−70	3	*t* = 63.70, *p* < 0.001	*t* = 76.45, *p* < 0.001
**SHAM > rTMS**							
10	L PMC BA6	2423	−31	−2	48	*t* = 39.80, *p* < 0.001	*t* = 20.94, *p* < 0.001
11	L IPL BA40 Cluster2	667	−41	−33	38	*t* = 31.93, *p* < 0.001	*t* = 16.68, *p* < 0.001
12	L IPL BA40 Cluster3	1103	−51	−41	41	*t* = 0.68, *p* = NS	*t* = −9.30, *p* < 0.001
13	L IPL BA40 Cluster5	650	−39	−52	45	*t* = −1.66, *p* = NS	*t* = −9.66, *p* < 0.001
14	L IPS BA39	1318	−33	−62	43	*t* = −0.02, *p* = NS	*t* = −10.1, *p* < 0.001
15	L Angular Gyrus BA39	2124	−34	−79	31	*t* = 16.64, *p* < 0.001	*t* = 5.41, *p* < 0.001
16	L MoG BA19	1069	−30	−84	18	*t* = 55.86, *p* < 0.001	*t* = 39.44, *p* < 0.001
**rTMS > SHAM**							
17	R MFG BA6	967	7	10	49	*t* = 25.48, *p* < 0.001	*t* = 38.54, *p* < 0.001
18	R IPL BA 40	3035	41	−39	48	*t* = 22.38, *p* < 0.001	*t* = 35.49, *p* < 0.001
19	R IPS	3178	32	−71	41	*t* = 3.63, *p* = NS	*t* = 21.43, *p* < 0.001
20	R Precuneus BA7 Cluster1	1309	11	−72	55	*t* = 14.46, *p* < 0.001	*t* = 26.95, *p* < 0.001
21	R Precuneus BA7 Cluster2	872	25	−81	42	*t* = 34.14, *p* < 0.001	*t* = 43.91, *p* < 0.001
22	R Precuneus BA31	719	19	−60	25	*t* = −19.00, *p* < 0.001	*t* = −8.77, *p* < 0.001
23	R Cuneus BA 18	4987	8	−71	11	*t* = −21.76, *p* < 0.001	*t* = −5.48, *p* < 0.001
24	R MoG BA19	743	29	−87	7	*t* = 16.08, *p* < 0.001	*t* = 27.47, *p* < 0.001
**SHAM > rTMS**							
25	R PMC BA6	1970	23	−2	51	*t* = 45.419, *p* < 0.001	*t* = 25.296, *p* < 0.001
26	R MFG BA6 Cluster1	985	39	−7	49	*t* = 56.827, *p* < 0.001	*t* = 39.885, *p* < 0.001
27	R IFG BA6	1119	44	4	24	*t* = 31.85, *p* < 0.001	*t* = 16.172, *p* < 0.001
28	R SPL BA 5	812	26	−40	54	*t* = 33.862, *p* < 0.001	*t* = 23.058, *p* < 0.001
29	R SoG BA19	632	23	−79	27	*t* = 47.460, *p* < 0.001	*t* = 34.959, *p* < 0.001
30	R Lingual Gyrus BA18	611	8	−75	−5	*t* = 45.970, *p* < 0.001	*t* = 31.715, *p* < 0.001
31	R MT	1482	44	−72	10	*t* = 50.504, *p* < 0.001	*t* = 34.403, *p* < 0.001

ROIs and their clusters, location (Talairach x, y, and z in mm) and intensities following concatenated runs (1, 2, and 3) of rTMS vs. sham. PMC, premotor cortex; M1 primary motor cortex; IPL, inferior parietal lobule; SPL, superior parietal lobule; IPS, intra parietal sulcus; MT, medial temporal; SoG, superior occipital gyrus; MoG, medial occipital gyrus; MFG, middle frontal gyrus; IFG, inferior frontal gyrus; AG, angular gyrus. Items 1–18: areas in the left hemisphere and items 17–31: areas in the right hemisphere.

**Table 2:**
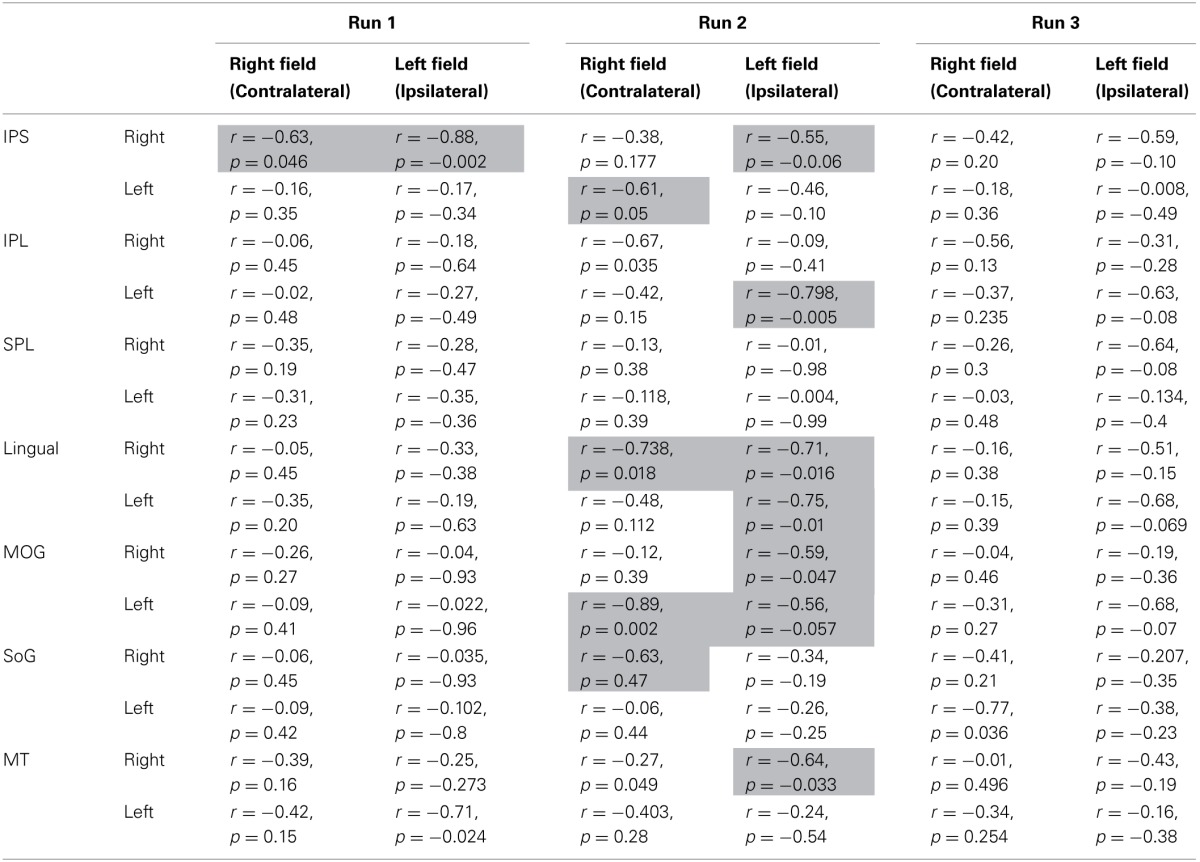
**Activation-accuracy relationship**.

Results of Pearson's correlation between normalized activation of a region (volume of activation following rTMS vs. sham) and normalized accuracy in a hemifield (accuracy following rTMS vs. sham). The relationships are presented for ROIs identified in multi-subject analysis. These ROIs were tested subject-by-subject and the resultant volume that was active following rTMS vs. sham was analyzed for correlation with their accuracy in the right and left hemifields. Significant correlations or trends toward significance have been highlighted in gray. Although a few other regions appeared to have a significant relation, for instance left MT with left field accuracy in Run 1, on close analysis, these correlations were removed as they were driven by individual outlier values.

### Summary of fMRI analyses and their relation to study hypotheses

The three levels of fMRI analysis discussed above align with our original hypotheses. We hypothesized that following rTMS of left IPS:

1. Multi-subject analysis, and comparison of intensities of homologous voxels of IPS would show reduced fMRI activation of targeted IPS with an opposite response of right IPS
2. Multi-subject analysis, and analysis of intensities of homologous ROIs would show that regions known as synergists of IPS in sustaining bilateral attention also demonstrate a shift in their inter-hemispheric activation analogous to the IPS
3. Nature of correlation between tracking accuracy and activation of IPS, and its network-wide synergists, would evolve in line with their role in sustaining bilateral attention.

## Results

### Behavioral task

Accuracy in both visual fields, contralateral and ipsilateral, was impaired immediately following rTMS (Run 1) (0.82 ± 0.10 and 0.93 ± 0.18, respectively). Relative to sham, the impairment tended to be considerable, albeit only approaching significance, for contralateral [*t*_(9)_ = 1.652, *p* = 0.066] but not in the ipsilateral field [*t*_(9)_ = 0.374, *p* = 0.35] (Figure 1C). For the 2nd run, accuracy in the contralateral field tended to improve but it was still reduced (0.92 ± 0.12). Relative to sham, however, its performance was not significantly different [*t*_(9)_ = 0.598, *p* = 0.28]. Also, although accuracy in the ipsilateral field resumed (1.1 ± 0.2), it was not significantly different relative to sham [*t*_(9)_ = 0.469, *p* = 0.325]. For the 3rd experimental run, accuracy in both ipsilateral and contralateral fields resumed to levels noted following sham (1.05 ± 0.19 and 1.01 ± 0.20, Figure 1C).

### fMRI results

Statistical parametric map (Figure 2) of the multi-subject fixed effects GLM analysis illustrated various ROIs that were differentially active following rTMS vs. sham across left and right hemispheres (Table 1 and Figure 2). We will first present results for multi-subject fixed effects GLM analysis, followed by analysis of homologous ROIs and activation-accuracy relationship for IPS. Later, we will describe these analyses for all other ROIs revealed with statistical parametric map.

#### IPS

Multi-subject fixed effects GLM analysis showed that left lateral IPS (BA 39) demonstrated lower activation following rTMS vs. sham, while in the right hemisphere, the pattern was reversed (Figure 2). When we compared voxels in left and right comprising the homologous IPS pair, we found that intensity of voxels on the left was significantly lower than ones on the right [*t*_(0.05,1169)_ = 139.71, *p* < 0.001] (Figure 3A).

Activation of IPS was related to behavioral accuracy in runs 1 and 2 only, but not in run 3. In particular, activation of right IPS was related to accuracy in the right hemifield (Figure 4A) and left hemifield in run 1 (Figure 4B), whereas in run 2, activation of left IPS was positively related with accuracy in the right hemifield (Figure 4C) and activation of right IPS was positively related with performance in the left hemifield (Figure 4D). In run 2, activation of left IPL was also significantly related to accuracy in the left field (Figure 4E).

#### Other ROIs

Network-wide ROI analysis (Figure 2) demonstrated that in the left hemisphere, parietal regions that lay medial to targeted (left) IPS, such as the Superior Parietal Lobule (SPL) (BA 5, 7) and the Precuneus (BA7), demonstrated higher activation following rTMS vs. sham, while those lying lateral and inferior to IPS, such as inferior-parietal lobule (IPL, BA40) showed lower activation. Occipito-temporal regions, Superior Occipital Gyrus (SoG) (BA19), Lingual gyri (BA18), Cuneus and Medial Temporal area (MT+) (BA37) showed higher activation, while Middle Occipital Gyrus (MoG) (BA19) demonstrated diminished response following rTMS vs. sham. In the right hemisphere, however, activation pattern of majority of these network-wide areas was reversed: SPL (BA5) demonstrated lower activation, while IPL showed higher activation following rTMS vs. sham (Figure 2). Similarly, SoG, Lingual and MT+ demonstrated lower activation, while MoG showed greater activation following rTMS vs. sham. Therefore, multi-subject analysis showed that following rTMS vs. sham, activation of IPL, SPL, SoG, Lingual, MoG and MT+ areas modulated at an inter-hemispheric level as well, analogous to IPS. The direction of modulation, however, i.e., increase in one hemisphere vs. decrease in another, varied.

The homologous ROI analysis confirmed findings of the multi-subject GLM (Figure 3). In comparing voxels commonly active in both hemispheres, we found that IPL, SPL (BA5), SoG, Lingual, MoG, and MT+ were affected at inter-hemispheric level as well similar to IPS (Figures 3A,B). Their intensities in TMS-targeted hemisphere were significantly different from those on the right. Intensity of voxels in the targeted left hemisphere was significantly lower than that of corresponding voxels in the right for IPL [*t*_(0.05,2910)_ = 106.81, *p* < 0.001] and MoG [*t*_(0.05, 709)_ = 64.8, *p* < 0.001], while intensity was higher on the left than right for SPL [*t*_(0.05,2278)_ = 86.2, *p* < 0.001], SoG [*t*_(0.05,1491)_ = 57.46, *p* < 0.001], Lingual [*t*_(0.05,1590)_ = 72.31, *p* < 0.001], and MT+ [*t*_(0.05,1322)_ = 57.87, *p* < 0.001]. Areas as cuneus and precuneus were not differentially modulated across hemispheres.

Different ROIs demonstrated varying relationships between their activation and the accuracy in left and right hemifields. These relationships evolved from one run to the next. Whereas activation of right IPS and its relationship to accuracy in left and right hemifields (Figures 4A,B) was significant in run 1, relationship between activation of other ROIs and accuracy only manifest for run 2. In run 2, contralateral (right hemifield) accuracy was positively related to activation of right Lingual (Figure 5A), left MoG (Figure 5C), and right SoG (Figure 5E), while ipsilateral (left field) accuracy was related to activation of right lingual (Figure 5B), left lingual (Figure 5D) and right MT+ (Figure 5F).

## Discussion

We used fMRI to determine whether rTMS over the left IPS directly alters inter-hemispheric interactions that may explain transient TMS-induced decrement in sustained visual attention, or extinction (Battelli et al., 2009). Our results suggest the following in relation to our original hypotheses. (1) After rTMS, activity of targeted IPS is lower while that of right IPS is exaggerated, reinforcing that inter-hemispheric balance of its activation is indeed disrupted with rTMS. (2) Besides IPS, inter-hemispheric balance of the network involved in bilateral sustained attention- parietal, temporal and occipital synergists- is also disrupted. IPL and MoG show lowered activation and SPL, MT+ and SoG show higher activation in TMS-targeted hemisphere, while response of their homologues is opposite. The transient attentional decrement induced with rTMS thus emerges from a network-wide disruption of inter-hemispheric balance. (3) The evolution of activation of IPS and its network-wide synergists relates to changes in attentional accuracy over serial runs; whereas, immediately, activation of right IPS is associated with right and left field accuracy, subsequently, synergists as IPL, Lingual gyrus, SoG, MoG, and MT+ likely relate to recovery. Although we did not find a strong decrement in behavioral performance, unlike our previous study, we noted with fMRI that network-wide activation of IPS, IPL and occipito-temporal synergists may help adaptively compensate for, and alleviate, contralateral and ipsilateral decrement after rTMS. Therefore, our model of combined rTMS and fMRI offers direct empirical demonstration of altered inter-hemispheric balance, a likely explanation of the clinical manifestation of visuospatial extinction, and the nature of such balance during attentional behavior.

### Intra-parietal sulcus (IPS): inter-hemispheric competition in bilateral visual attention

IPS is implicated in resolving competition between bilateral stimuli (Culham et al., 1998; Muri et al., 2002; Muggleton et al., 2006; Battelli et al., 2009), an ability that emerges from tonic inhibitory influence exerted by one IPS upon another (Kinsbourne, 1977). That rTMS targeting IPS intensifies this inter-hemispheric competition has traditionally been inferred from behavioral observations (Hilgetag et al., 2001; Muri et al., 2002; Thut et al., 2005; Dambeck et al., 2006; Fierro et al., 2006; Battelli et al., 2009). Here, we demonstrate for the first time that TMS targeting left IPS indeed reduces activity of left and increases activity of right IPS. TMS likely weakens inhibition exerted by left upon right IPS, which in turn is disinhibited. As a novel finding here, with the use of fMRI measurement of offline effects of rTMS, we generate empirical support for the theory of inter-hemispheric rivalry, shedding new light on the basis of clinically witnessed extinction-like effects (Kinsbourne, 1977; Corbetta et al., 2005).

Interestingly, increased activity of right IPS relates positively with accuracy in hemifields ipsilateral as well as contralateral to targeted IPS. While the former finding aligns with the belief that uninhibited activity of “undamaged” parietal cortex leads to hyper-oriented attention to the unimpaired field, the latter is in contradiction to its corollary. Hyper-oriented attention to the unaffected field is long-thought to limit attention to the impaired field even further.

We however have failed to observe a “negative” effect of over-activation of right IPS upon right-field attention potentially for the following reasons. First, we elicited weaker right-field extinction with rTMS in our present study compared to our previous (Battelli et al., 2009). The time lapse between end of rTMS and beginning of behavioral task in the scanner could have mitigated the impairment, which may have affected over-activation of right IPS, hence its ability to hyper-attend to left and limit attention to right field. Second, since right IPS is specialized to subtend visual attention in both hemifields (Mesulam, 1981), its disinhibition may in fact have a compensatory role- alleviating rTMS-induced decrement in right-field visual attention. Therefore, “disinhibition” of right parietal cortex, since it subtends attention to left as well as right fields, may have led to a milder level of right-field extinction following inactivation of the left parietal cortex. Understanding whether inactivating right IPS exaggerates the right-field decrement would be important to confirm our speculation. Thus, fMRI combined with offline rTMS to unilateral left IPS generates empirical support for the theory of inter-hemispheric rivalry (Corbetta et al., 2005).

### Inter-hemispheric competition across network involved in bilateral visual attention

Although IPS was the target locus, activity of several important parieto-occipito-temporal synergists modulated differentially across both hemispheres indicating that rTMS of left IPS influences inter-hemispheric balance of the entire visuo-attentional network. Even more importantly, our model combining offline fMRI following rTMS demonstrated that inter-hemispheric profile of activation of these synergists aligns with the nature of their interaction with IPS in sustaining bilateral visual attention. Whether the activation of a region was in line with or opposite to that of targeted IPS indicates its role in supporting visual attention (Sheremata et al., 2010). For instance, IPL's inter-hemispheric activation pattern coincides with that of the IPS, suggesting their paired role in visual attention (Cicek et al., 2007) and involvement with visuo-spatial neglect (Mort et al., 2003). On the other hand, SPL and Precuneus show opposite inter-hemispheric activation pattern than IPS and IPL, which reinforces the theory of dynamic competition between these pairs. Medial-dorsal regions, such as SPL and Precuneus, are anatomically segregated and functionally competing in a push-pull manner with lateral-ventral parietal regions, as IPS and IPL (Sestieri et al., 2010). While SPL and Precuneus trigger transient attentional shifts to bilateral loci, IPS and IPL are involved in sustaining attention to both visual fields (Battelli et al., 2001; Kelley et al., 2008). Thus, while IPS and IPL showed a similar inter-hemispheric response to rTMS, SPL, and precuneus demonstrated the opposite with their respective homologues (Figures 2, 3A).

Occipital synergists showed varying inter-hemispheric response as well, in line with the nature of their relation with IPS. We witnessed opposing responses of SoG from the IPS- exaggerated facilitation on left, with inhibition on right, following rTMS. Activation of early visual areas as SoG is functionally coupled yet dynamically competing to that of posterior parietal regions (Ruff et al., 2008), which would explain their contrasting response to rTMS. Dynamic interactions were not only visible between homologous pairs, but also between non-homologous synergists. For instance, left MoG and right lingual showed opposite response to rTMS; while lingual became more active in the left hemisphere, MoG became more active in the right following rTMS. MoG and lingual in different hemispheres maintain a competitive dynamic that has been described previously in the context of visual motion perception (Brandt et al., 2000). The presence of a contralateral moving visual stimulus has been associated with MoG activation that is paired with that of ipsilateral lingual gyrus. Such an inverse relation between non-homologous regions as that modulated in our protocol is believed to arise from transcallossal transfer of visual attention information (Brandt et al., 2000).

It is finally interesting to notice the temporal evolution of activation across regions. How the activation of a region evolves with attentional behavior indicates what type of role it exerts in visual attention. Immediately following rTMS, intensity of activation of right IPS was positively related to that of accuracy in contralateral (right) and ipsilateral fields. In the 2nd run, participants showing higher activation of left MoG and right Lingual showed higher right-field accuracy, indicating inter-hemispheric interactions of these non-homologous occipital synergists may serve to adaptively compensate for rTMS-induced deficits.

Overall, thus, empirical use of fMRI with offline rTMS directly supports Kinsbourne's hemispheric rivalry in bilateral sustained visual attention, suggesting its potential link to clinical visual extinction. We have noted that empirical visual extinction induced by rTMS to IPS is subtended not only by an inter-hemispheric imbalance at the level of IPS, but also its functional network involving parietal, temporal and occipital synergists. Competitive inter-hemispheric profile was witnessed for IPL, SPL, SoG, MT+, MoG besides IPS. Following rTMS, the similarity between the response of a synergist and that of targeted IPS, and the evolution of such response, alludes to the nature of their mutual interactions in bilateral visual attention- competing or compensatory. While competitive coupling is noted for IPS/IPL vs. SPL/Precuneus and IPS vs. SoG, adaptive interactions of MoG and Lingual gyrus with IPS may help alleviate behavioral decrement. Therefore, our model combining rTMS and fMRI offers direct empirical insight into altered coupling believed to explain clinical phenomena, and the nature of such coupling in the performance of normal behavior.

## Conclusions

Using a protocol of offline rTMS combined with fMRI, we studied network-wide mechanisms of rTMS targeting IPS in bilateral sustained visual attention. We showed proof-of-concept for classical theory of hemispheric rivalry that manifests in bilateral attention, by showing competing activation between hemispheres across areas critical to visual attention. Further, by illustrating intra- and inter-hemispheric interactions with the targeted locus, we suggest transient compensatory phenomena that could attenuate the behavioral effects of inactivating IPS. Such intra- and inter-hemispheric connectivity empirically supports the clinical extinction noted with damage to posterior parietal cortices and workings of distributed neural systems that potentially favor recovery from focal damage. Finally, these findings have important implications for potentially using rTMS as a rehabilitation technique for severe and persistent attentional deficits following parietal stroke.

## Conflict of interest statement

The authors declare that the research was conducted in the absence of any commercial or financial relationships that could be construed as a potential conflict of interest.
